# Evaluation of the Colour Rendering of Brand Identity Elements on Sustainable Papers Made from Invasive Alien Plant Species

**DOI:** 10.3390/jimaging12050193

**Published:** 2026-04-30

**Authors:** Anja Sarjanović, Klemen Možina

**Affiliations:** Department for Textile, Graphic Arts and Design, Faculty of Natural Sciences and Engineering, University of Ljubljana, Aškrčeva cesta 12, SI-1000 Ljubljana, Slovenia; anja.sarjanovic.student@gmail.com

**Keywords:** Japanese knotweed, sustainable printing, invasive alien species, colour reproduction, ICC profiles, colour differences, inkjet printing, electrophotography, brand identity

## Abstract

The use of invasive plant species for papermaking presents both environmental and economic opportunities, particularly for companies seeking to introduce sustainable materials. This study examined whether paper made from cellulose fibres of Japanese knotweed is suitable for printing business elements such as logos in specific red colours. The physical, mechanical, and optical properties of the paper were compared with those of standard office and commercial Xerox paper. Two printing techniques—electrophotography and inkjet printing—were tested, and the colour differences (CIE colour difference, ΔE) between the reference logo and the prints, with and without the International Colour Consortium (ICC) colour profile, were evaluated. The results showed that the low whiteness and high porosity of the knotweed paper negatively affected colour reproduction, especially in inkjet printing, where even manually optimised profiles did not yield satisfactory results (minimum ΔE > 23). Electrophotography performed better but still had limitations. It was concluded that Japanese knotweed paper is not suitable for professional reproduction of demanding colour elements without additional processing, although it has potential for sustainable applications with lower visual requirements.

## 1. Introduction

Invasive plants are among the greatest challenges facing ecosystems today [1]. Their impacts include habitat modification, changes in soil composition, interference with native plant species, and reduced biodiversity [2,3,4,5]. Due to their widespread distribution and resilience, invasive species pose significant ecological and economic problems worldwide, necessitating effective containment strategies and innovative reutilisation methods [6,7,8]. Japanese knotweed (*Fallopia japonica*) is one of the most widespread invasive alien species [9,10]. Eradicating Japanese knotweed and similar species is a lengthy and often unsuccessful process [5,9,11].

In recent years, interest has grown in converting this plant into useful products as part of sustainable biomass utilisation strategies [6,7]. One promising solution is the use of its cellulose fibres for paper production [5,12,13,14]. Knotweed is an inexpensive raw material, but its processing requires specific adaptations to achieve suitable printing properties [15,16].

Previous studies suggest that papers made from invasive plants may be mechanically and optically suitable for printing [12,14,17]. The samples showed sufficient cellulose content and physical properties comparable to those of conventional pulp papers [12,13]. However, some challenges remain– especially in printing– due to factors such as fibre type, chemical composition, porosity, surface roughness, whiteness, lignin content, and mechanical behaviour [15,16,17,18,19]. An accurate assessment of printability requires precise knowledge of the optical, mechanical, and surface properties of the paper [18,20].

Studies have shown that knotweed paper generally exhibits higher roughness and porosity, and a lower degree of whiteness, which results in ink bleeding and reduced print contrast, particularly with wet printing techniques such as inkjet printing [5,15,21,22]. In contrast, dry techniques such as electrophotography are more suitable for these papers, as they reduce the risk of deformation caused by ink absorption [5,23].

Colour reproduction on these materials presents a particular challenge. A key factor is the consistency of colour values between digital designs and the final print. ICC (International Colour Consortium) profiles facilitate colour management across devices, but their effectiveness on papers with non-standard optical properties has not yet been thoroughly investigated [24,25]. It is known that higher paper whiteness contributes to greater colour saturation and contrast [21,23,26,27,28]. Conversely, a lower degree of whiteness results in greater colour differences (ΔE), which impair the perception of brand elements such as logos [22,26].

Paper tends to yellow over time, affecting both the appearance and durability of the print due to photo-oxidative and chemical ageing processes [14,15,16]. Therefore, it is important to understand the effects of ageing and mechanical stress on the durability of prints when using alternative papers such as knotweed-based substrates. Previous findings suggest that UV-exposed knotweed paper reacts differently from commercial paper in terms of its mechanical properties, potentially impacting its long-term usability [15].

Despite existing studies, it has not yet been thoroughly investigated whether certain graphic elements, such as company logos, can be reproduced on Japanese knotweed paper. This represents a critical research gap, as colour accuracy is crucial for brand recognition and the professional appearance of printed materials.

The aim of this study was to answer the following question: How accurately can challenging colours—especially dark red—be reproduced on Japanese knotweed paper, and what techniques or processes can improve reproduction quality? To address this, an experimental comparison was conducted of three paper types (standard office paper, Xerox Colotech, and knotweed paper), two printing techniques (electrophotographic and inkjet), and different methods of colour profiling (custom ICC profiles, saturation tests, and use of industry profiles). The objectives of the study were to:

- Compare the physical and optical properties of the papers;
- Evaluate the effects of printing techniques on the colour consistency of the logo;
- Test the effectiveness of the different ICC profiles and manual settings;
- Correlate the ΔE values with the paper properties and determine the realistic limits of knotweed paper in professional printing.

## 2. Materials and Methods

### 2.1. Research Process

Given the remarkably low degree of whiteness and the pronounced absorption properties of paper made from Japanese knotweed, we addressed the question: Can colour profiles, printer settings, or CMYK value adjustments improve colour reproduction on this paper? The investigation was conducted in five phases:

1. Measurement of the paper properties;
2. Testing of basic printing technologies without colour management;
3. Development and use of a custom ICC colour profile for inkjet printing;
4. Analysis of the influence of colour saturation and coverage on ΔE;
5. Profile optimisation and testing with existing ICC standards.

Although the experimental work was conducted in cooperation with a company with a red and grey logo, the broader objective of this study was to evaluate the general suitability of knotweed paper for reproducing saturated corporate colours that require high chromatic accuracy.

### 2.2. Paper Properties Measurement

Three types of paper were compared: paper made from the invasive alien plant Japanese knotweed (hereinafter referred to as knotweed paper), commercial Xerox paper, and standard office paper. The knotweed paper was produced on a pilot paper machine from Andritz AG (Graz, Austria), installed at the Institute for Pulp and Paper in Ljubljana. The raw material consisted of a mixture of Japanese knotweed (40%), spruce (20%), and eucalyptus fibres (40%). Before the printing tests, the basic properties of all three paper types were analysed. The samples were conditioned under standard environmental conditions of 23 °C and 50% relative humidity, in accordance with ISO 187:2022 [29].

#### 2.2.1. Structural Properties of Paper

##### Grammage, Thickness, Density, and Specific Volume

The grammage was determined in accordance with ISO 536:2019 [30]. The thickness, defined as the third dimension of the paper or the perpendicular distance between the surfaces, and the specific volume were measured in accordance with ISO 534:2011 [31], as were the density and specific volume. Thickness was measured with a Mitutoyo micrometre with an accuracy of 0.001 mm.

##### Moisture Content

The samples were exposed to standard climate conditions (23 °C and 50% RH) in accordance with ISO 287:2017 [32]. The moisture content, which significantly influences properties such as mechanical strength, dimensional stability, drying time, and printability, was determined gravimetrically by weighing the sample before and after drying at 105 °C.

#### 2.2.2. Surface Roughness and Porosity

Surface properties were assessed by measuring roughness and air permeability according to the Bendtsen method for roughness and for determination of air permeance using the Bendtsen N3500 instrument (PTE – Pulp Test Equipment GmbH, Eberstalzell, Austria) [33].

#### 2.2.3. Mechanical Properties: Sound Velocity, Elasticity, and Stiffness

The mechanical properties depend on the morphological properties of the cellulose fibres and the parameters of the paper manufacturing process. Stiffness was determined using the Clark method. Although not standardised, Clark stiffness (CS) is a valuable indirect indicator of the bending stiffness of paper, which is particularly relevant for printing substrates. Paper strips (25 mm wide) were exposed to a sound pulse of 160 Hz at a measurement interval of 1 cm using the Pulse Propagation Meter PPM-5R (Lawson-Hemphill Inc., Swansea, MA, USA).

Sound velocity (C) was calculated as:

(1) $$C=\frac{∆l}{∆t}$$

where Δl is the distance between transmitter and receiver [cm], and Δt is the signal travel time [μs].

Young’s modulus of elasticity (E) was then calculated as:

(2) $$E=\rho \times C^{2}$$

where ρ is paper density [kg/m^3^].

Clark stiffness (CS) was calculated as:

(3) $$CS=\frac{E\;\times \;d^{3}}{12}$$

where d is the paper thickness [mm].

#### 2.2.4. Optical Properties

##### Gloss

The gloss was measured in accordance with ISO 8254-1:2009 [34] using a 75° gloss meter. Measurements were taken on both sides of the paper (A and B) and in both the machine direction (MD) and cross direction (CD).

##### Whiteness

The ISO whiteness was determined according to ISO 11475:2017 [35]. The colour coordinates in CIELAB space were measured according to ISO 5631-1:2022 [36] using an X-Rite i1 spectrophotometer (x-rite Pantone, Grand Rapids, MI, USA) with a geometry of 45°a:0°, D65 illumination and a 10° standard observer.

#### 2.2.5. Ageing Resistance

Artificial ageing tests were conducted to evaluate pressure resistance by exposing the samples to xenon light in a Xenotest^®^ Alpha (Atlas Material Testing Technology, Mount Prospect, IL, USA) chamber in accordance with ISO 12040:1997 [37]. Chamber conditions were 35 °C, black panel temperature 50 °C, and 35% relative humidity. The exposure lasted three days, simulating approximately six months of natural light.

#### 2.2.6. Rubbing Resistance

Rub fastness was evaluated with the Param RT-01 Rub Tester under loads of 12 N and 24 N. Colour fastness after rubbing was assessed visually and spectrophotometrically.

### 2.3. Influence of Colour Management on the Quality of Colour Reproduction

Several consecutive experiments were conducted to evaluate the influence of paper, printing technique, and colour profile.

#### 2.3.1. Printing and Sample Preparation

Both printing techniques were used on all three types of paper:

- Electrophotography: Xerox Versant 280 Press (XEROX, Norwalk, CT, USA) (production digital press);
- Inkjet: Epson WorkForce Pro WF-C579R (Epson, Nagano, Japan) (office inkjet printer).

Electrophotographic printing (EP) is a dry digital printing technique based on electrostatic image formation and toner transfer. The process involves several consecutive steps: (i) uniform electrostatic charging of a photoconductive drum, (ii) selective discharge of the drum by a laser or LED exposure system according to the digital image, forming a latent electrostatic image, (iii) development, in which finely powdered toner particles are attracted to the charged image areas, (iv) transfer of the toner image onto the paper substrate by electrostatic forces, and (v) thermal and pressure fusing, where the toner is permanently fixed to the paper surface. Unlike inkjet printing, electrophotography does not use liquid inks or rely on absorption into the paper structure, but instead deposits colour primarily on the surface of the substrate. This makes the technique less sensitive to high porosity, surface roughness, and moisture content of paper, which is particularly relevant for papers made from invasive alien plant species. As a result, electrophotography generally provides more stable colour reproduction and higher consistency on non-standard and uncoated papers with low whiteness, as confirmed by the lower colour differences (ΔE) observed in this study.

**Figure 1:**
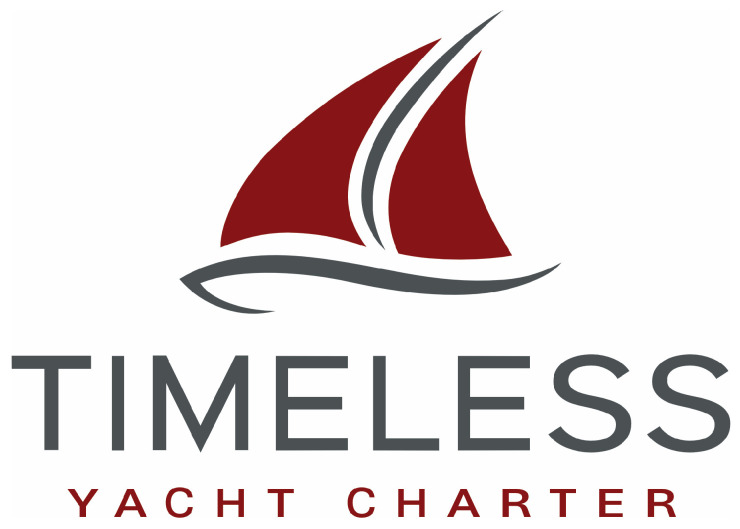
The reconstructed company logo.

Before printing, the logo was recreated in vector format using Adobe Illustrator CC 2023, version 27, to ensure compliance with ISO 16612-2:2010 [38] for vector graphics (Figure 1). As precise colour specifications (such as brightness or exact channel values) were unavailable, approximately dark shades of red and grey were selected to match the original design (CMYK: red = C25 M100 Y100 K35; grey = C30 M20 Y20 K70).

#### 2.3.2. Testing Primary Colour Tones with Varying Saturation

Colour fields were printed for the primary colours (cyan, magenta, yellow—CMY) and for combinations representing specific hues such as red and grey, which form the logo of DTM Tehnologije d.o.o. For each hue, the saturation of the individual channels was systematically varied, and colour patches were printed and then evaluated spectrophotometrically. The measured ΔE values were compared with the vector logo template to determine the smallest achievable colour differences and to identify combinations that most closely matched the original colour values on the Japanese knotweed paper.

The colour difference ΔE was calculated in the CIELAB colour space, where L* represents lightness, a* the red–green coordinate, and b* the yellow–blue coordinate:

(4) $$∆E=\sqrt{\left({\left(L_{1}^{*}-L_{2}^{*}\right)}^{2}\;+\;{\left(a_{1}^{*}-a_{2}^{*}\right)}^{2}\;+{\left(b_{1}^{*}-b_{2}^{*}\right)}^{2}\right)}$$

(ISO/CIE 11664-6:2014 [39]).

After printing, each combination of paper and printing technique was visually assessed and photographed, followed by quantitative measurements of colour consistency. Measurements were taken using an X-Rite i1Pro 3 spectrophotometer and i1Profiler 3.6.1 software (in accordance with ISO 13655:2017 [40]). The measurement colour space was CIELAB (ISO 11664-4:2019—Colorimetry [41]), and the ΔE values were calculated according to ISO 12647-7:2016—Colour difference metrics [42].

#### 2.3.3. Testing Without Colour Management

In the first phase of the colour difference measurements, no colour profiles or colour management systems were used to create the print files.

#### 2.3.4. Development of a Customised ICC Profile for Inkjet Printing

In the second phase, a new ICC profile for inkjet printing on Japanese knotweed paper was developed in accordance with ISO 15076-1:2010—Colour profiles for colour management [43]. The software used was i1Profiler 3.6.1, compliant with ISO 13655:2017 [40]. The profile was left at the default settings to observe the results under baseline conditions. The only change was the number of colour patches on the test card, which was set to 700. Due to the very poor results of the colour profile created and the corresponding print, it was assumed that an error had occurred during profile creation. When an attempt was made to reopen and correct the profile, the software displayed excessive deviations between the captured colour patches. After several unsuccessful attempts to recreate the profile, the experiment was temporarily abandoned in favour of alternative approaches aimed at reducing the colour difference to at least a ΔE value of 5.

#### 2.3.5. Analysis of the Effects of Colour Saturation and Coverage

To better understand the influence of different colour tones on print quality, a set of fifty colour tones was created from six primary colours: red, grey, black, blue, green, and yellow. All samples were printed on three types of paper using both printing techniques, inkjet and electrophotography. The colour difference (ΔE) was then measured for each hue, providing information on how accurately each colour was reproduced compared to its reference hue.

#### 2.3.6. Profile Optimisation and Testing with Existing ICC Standards

Based on the results, the next step was to manually adjust certain parameters in the inkjet printer’s color profile. An additional attempt was made to print the sample using ICC profiles that are already available and widely used in the market. Two custom profiles were created to investigate how adjustments to the profile parameters affect colour reproduction on the paper. All profiles in this study were created using i1Profiler software in conjunction with the X-Rite i1Pro 3 spectrophotometers.

Settings for the first profile (all other settings remained at the default):

- Black start: 50
- Contrast: 50
- Saturation: 50
- Neutralise grey: 25
- White point: 77.8: 5.0: 19.0

Settings for the second profile (all other settings remain at the default):

- Increased black generation
- CMY ratio: 50
- Contrast: 25
- Saturation: 50
- Neutralise grey: 0

Another profile was created for Xerox paper with inkjet printing, with all settings left at default. The resulting print was compared with the original print on Xerox paper. The commercial profiles tested to achieve the best possible colour match were:

- ISO uncoated yellowish
- Japan Colour 2002 newsprint
- Fogra 39

## 3. Results

### 3.1. Paper Properties

#### 3.1.1. Thickness, Grammage and Density

Although the basis weight of knotweed paper and Xerox paper was similar, the difference in thickness was considerable. Knotweed paper had the greatest average thickness, indicating a significantly higher volume-to-weight ratio. This was also reflected in its lowest measured density, suggesting a less compact, more porous structure. In contrast, Xerox paper had the highest density and the lowest thickness, indicating a more compact and dense structure. Office paper occupied an intermediate position for these parameters. The lower density of knotweed paper may be due to specific fibre properties, such as shorter fibre length, higher void content, or a lower degree of processing (e.g., less calendaring, higher lignin content). These physical differences strongly influence the mechanical and printing properties of the paper. The greater thickness and lower density of knotweed paper can affect stiffness and bending behaviour, which are important for further processing during printing. The measured values for thickness, grammage, and density of the tested papers are listed in Table 1.

#### 3.1.2. Moisture Content and Specific Volume

The specific volume of the papers reflects the mass-to-volume ratio and confirms the results from the previous section. Knotweed paper occupies the largest volume per unit mass, consistent with its greater thickness and lower density. Xerox paper has the lowest specific volume, confirming its high compactness, while office paper falls between these two values. The moisture content is also notable. Knotweed paper contains almost twice as much moisture as office paper and Xerox paper. This difference is probably due to the more open structure of the paper, which allows greater absorption of water vapour from the environment, as well as the less processed nature of the fibres. Higher moisture content can negatively affect dimensional stability, mechanical properties and, above all, behaviour during the printing process, where deformation or poor ink transfer may occur. For applications in precision printing processes, additional drying or surface treatment of knotweed paper would therefore be advisable. The measured values for moisture content and specific volume of the tested paper grades are listed in Table 2.

#### 3.1.3. Surface Roughness and Porosity

Xerox paper had very low surface roughness and lower porosity due to mechanical surface smoothing and optimisation for printing. Office paper was significantly rougher and more porous, which affects ink absorption and final print quality. The measured values for surface roughness and porosity of the tested paper grades are shown in Table 3.

#### 3.1.4. Mechanical Properties: Speed of Sound, Elasticity and Stiffness

Xerox paper had the highest values for the speed of sound and the highest modulus of elasticity, indicating that it is the least flexible and stiffest material among the papers tested. Office paper and knotweed paper showed similar sound velocities in the machine direction but differed significantly in other mechanical parameters. Japanese knotweed paper stood out in terms of Clark stiffness (CS), achieving the highest values in both the MD and CDs. This indicates that, despite its lower elasticity (E), it has higher flexural strength due to its greater thickness. In practice, this results in greater stability during manual handling, folding, or mechanical processing. The measured values for speed of sound, modulus of elasticity, and Clark stiffness for the tested paper grades are listed in Table 4.

#### 3.1.5. Optical Properties: Specular Gloss

Xerox paper exhibited the highest specular gloss (up to 10.4% in the CD), which is typical of surfaces that have been heavily calendared and mechanically treated. Knotweed paper showed slightly lower gloss values (up to 5%), but these were still higher than those of office paper, indicating that its surface is not completely rough. Lower gloss generally results in a duller appearance and greater light absorption, which can affect print contrast and visibility. Surface gloss plays an important role in the visual perception of colours and print sharpness, especially in techniques based on the surface deposition of dyes. The measured gloss values for both sides (A and B) of the tested paper grades in the machine direction (MD) and cross direction (CD) are listed in Table 5.

#### 3.1.6. Optical Properties: Whiteness

When measuring the degree of whiteness using the R457 D65 method and the CIE W index, clear differences were observed between the papers. Xerox achieved the highest whiteness value, which corresponds to its intended use in high-contrast printing applications. Office paper had a slightly lower value, while knotweed paper had a very low whiteness value, reflecting the natural colour of its fibres and the absence of bleaching agents. The CIE W value for knotweed paper also confirms its darker, yellowish-brown appearance. The lower whiteness limits the use of knotweed paper in applications where high colour contrast or accurate colour matching is important, such as business stationery. The measured whiteness values for the tested paper grades are listed in Table 6.

#### 3.1.7. Ageing of the Prints

Colour differences after ageing were measured on the printed red logo area using CIE ΔE*00 values. The results indicate that the tested paper grades exhibit different levels of colour stability depending on the printing technique. According to ISO 12647-7:2016 [42], tolerances and industry interpretation of ΔE*00 colour differences, average colour differences in proofing workflows are typically limited to approximately ΔE00 ≤ 2.5, while values below ΔE00 ≈ 1.0 are generally considered imperceptible and values around 1–2 are often regarded as visually acceptable under controlled viewing conditions.

The greatest changes were observed in inkjet printing, indicating lower durability of this technique for all three papers. The worst result in this segment was recorded for Xerox paper, possibly because it was optimised for the electrophotographic technique and not for the water-based inks used in inkjet printing. Knotweed paper performed better in inkjet printing but showed slightly higher colour differences in electrophotography due to its pronounced roughness and porosity, which prevent uniform toner deposition. Office paper performed relatively evenly with both techniques. These results confirm how important it is to match the paper to the printing technique and the conditions of use. While knotweed paper can maintain relatively good colour stability in inkjet printing, its more porous structure and lower whiteness limit its use in demanding applications that require vivid and durable colours, especially when exposed to light. The measured colour differences after ageing for the tested paper grades and printing techniques are listed in Table 7.

#### 3.1.8. Rubbing Resistance

The abrasion resistance test was conducted with both printing techniques; however, relevant results were obtained only for inkjet printing. The knotweed paper showed almost identical colour change values at both low (12 N) and high (24 N) loads, indicating that the amount of force applied does not significantly affect the durability of the ink—the pigments are easily removed regardless of the force applied. At higher loads, the office paper performed best, while Xerox had the lowest rub resistance. These differences highlight the important role of surface structure and possible coatings in protecting the ink layer. For applications where mechanical resistance is crucial (e.g., packaging, envelopes, frequently handled materials), knotweed paper is less suitable due to its open structure and low surface protection, unless additional treatments (varnishes, coatings) are applied. The measured colour change values (ΔE) after rubbing for the tested paper grades are listed in Table 8.

### 3.2. Logo Reproduction and Colour Differences (ΔE)

#### 3.2.1. Initial Tests of Printing Technologies Without Colour Management

In the first phase of the study, the performance of paper made from Japanese knotweed with different printing technologies was investigated under conditions without colour management. The results showed clear differences in the quality of colour reproduction between the two technologies. Electrophotographic prints on all three papers achieved a very good colour match with the reference vector logo. The measured colour differences (ΔE), which quantify the deviation between the printed and reference colours, ranged from 1.92 to 8.04, which is considered acceptable according to ISO 12647-7:2016 [42]. These values fall within the range of acceptable differences, where deviations are visible to the average viewer but not overly disturbing. In contrast, the results of inkjet printing showed significantly poorer colour reproduction. Very high ΔE values were measured for all paper types, indicating extremely large colour differences (ΔE = 25–68), which are unacceptable for professional printing. Visually, this resulted in clearly faded printouts in which the red component was weak and almost completely absent. These results show that, without appropriate colour management, inkjet printing is unsuitable for the precise reproduction of demanding graphic elements, such as logos with defined colour standards. The final appearance of the print–and the printed colours—was also strongly influenced by the type and specifications of the substrate used. In this case, the Japanese knotweed paper, with its brownish-yellow hue, caused the printed colours to appear warmer, slightly shifted towards the yellow spectrum, and less contrasting compared to white paper. As the inkjet technique does not completely cover the substrate, inkjet printing with a white underlayer would be the best way to achieve a more accurate target colour in this case. The colour difference from the original resulted in a value of ΔE = 26.02. At this point, it was already clear that standard print settings would not be sufficient for satisfactory colour reproduction on Japanese knotweed paper and that colour management would be required.

#### 3.2.2. Development of a Customised ICC Profile for Inkjet Printing

Due to the inadequate results obtained without colour management, an individual ICC profile for inkjet printing on Japanese knotweed paper was created in the second phase of the study. The preview images on the screen already showed considerable deviations when the colour patches were captured. The final print on knotweed paper appeared pale violet, confirming the inadequacy of the profile settings. At the measurement level, ΔE was 43.37, confirming the counterproductive effect of the profile. The data for the red colour deviation were as follows:

- Red profile: L* 59.59, a* 10.83, b* 11.35;
- Red original (Xerox–electrophotography): L* 29.35, a* 39.63, b* 23.07;
- ΔE = 43.37.

**Figure 2:**
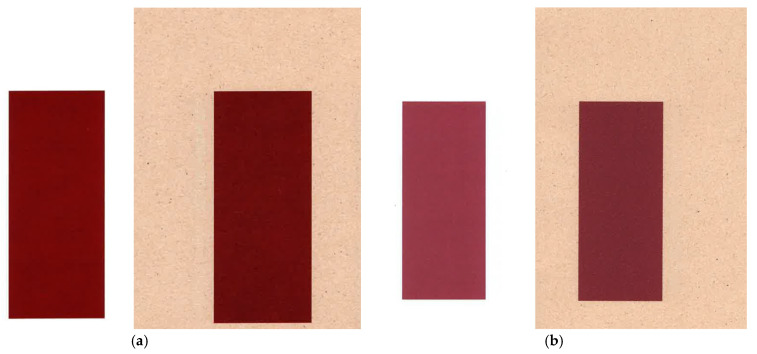
(**a**) Red with the applied profile used (inkjet printing); (**b**) desired red (electrophotography).

Figure 2a shows the red colour printed with the applied profile (inkjet printing), while Figure 2b shows the desired red obtained by electrophotography. These images visually confirm the significant deviation, with the red produced with the profile showing a muted, purple-shifted tone compared to the vibrant target colour.

The deviation of the red produced with the profile from the original red is shown in Table 9.

#### 3.2.3. Analysis of the Effects of Colour Saturation and Coverage on ΔE

In the third phase of the study, a more detailed analysis was conducted to examine how different degrees of saturation and opacity of the primary colours (CMYK) are reflected in colour differences between various papers. Fifty shades of the primary colours were prepared, ranging from very low to full coverage. The best result for the red colour was obtained with ΔE = 23.45, which remains a considerable deviation. Overall, empirical values show that inkjet printing has greater difficulty reproducing darker and more saturated colours compared to electrophotography. Inkjet printing does not achieve the same depth and intensity of colour tones. This is due to both technical limitations and the paper’s ability to absorb larger amounts of ink. An interesting observation was made with black. Contrary to the expectation that black would show the least variation, this was not necessarily the case. The best result was achieved with the colour tone where all four base values (CMYK) were set to 100%, giving a total coverage of 400%. The colour difference between the print on office paper and that on paper with Japanese knotweed in this case was ΔE = 3.79, which indicates a very good match.

However, such a high degree of coverage brings further challenges. Paper printed with a coverage of 400% becomes susceptible to physical deformation. Printing on both sides can lead to over-moistening of the surface, which negatively affects the structure of the paper. This makes it more fragile, less absorbent, increases drying times, and ultimately impairs the legibility of the printed product. When analysing the other colours—blue, red, and green—significant differences were also found between the different types of paper. The smallest and largest measured colour deviations (ΔE) between office paper and Japanese knotweed paper were as follows (ISO 12647-7:2016—colour difference metrics [42]):

- Blue: ΔEmin = 13.27, ΔEmax = 68.77;
- Red: ΔEmin = 8.10, ΔEmax = 64.15;
- Green: ΔEmin = 3.32, ΔEmax = 59.40.

**Figure 3:**
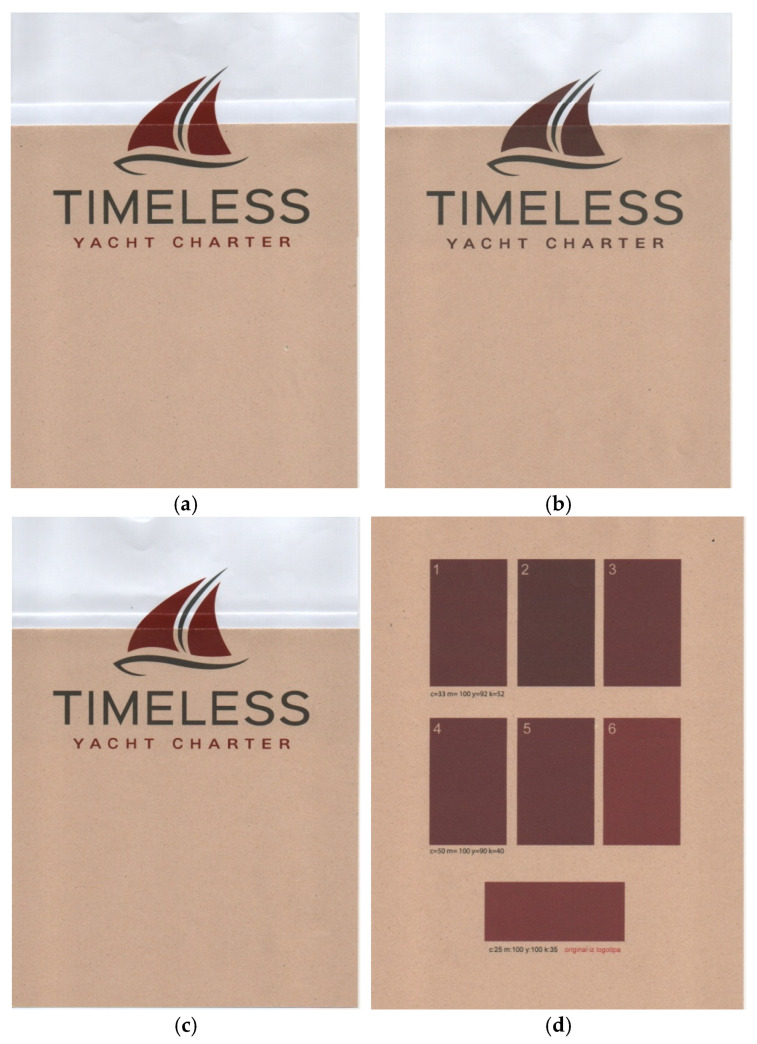
(**a**) Comparison of prints (electrophotography—original print); (**b**) comparison of prints (inkjet print—original print); (**c**) comparison of prints (electrophotography—best approximate colour value); (**d**) comparison of the best prints of approximate red tones from inkjet printing on Japanese knotweed paper.

These results show that the differences between the materials and printing processes are very pronounced and often exceed what would be acceptable for high-quality colour printing. A detailed analysis of the results confirmed the hypothesis already established in the analysis of the black colour: the higher the saturation or coverage of the printed area, the smaller the colour difference between the compared samples. This means that more saturated colours allow for greater consistency, regardless of the chosen paper or printing technique, although the physical limits of the materials must also be considered. The visual comparisons of the prints for both printing techniques and for the best colour approximations are shown in Figure 3.

#### 3.2.4. Optimisation of the Profile and Testing with Existing ICC Standards

In the final phase of the study, it was examined whether manual adjustments or existing ICC profiles could improve the colour matching of the logo on Japanese knotweed paper. Using manually adjusted profiles, it was found that changes to parameters such as contrast, grey neutralisation, and black emphasis did not yield the desired results. The first adjusted profile produced a washed-out image, with the red component noticeably shifted towards violet. The second profile, which increased the proportion of black, improved contrast but resulted in dark prints and thus lower colour accuracy. In contrast, the best result from all our approaches was achieved with a profile using the default settings on Xerox paper, producing a colour difference in ΔE = 27.78 (Table 10). Although this result remains above the recommended threshold for accurate reproduction (ΔE < 5), it demonstrates that, under certain conditions, default settings can provide better results than manually adjusted profiles that are not fully matched to the substrate’s properties.

## 4. Discussion

The results of the study confirm that paper made from cellulose fibres of Japanese knotweed presents a major challenge for accurate colour reproduction in business applications due to its specific physical and optical properties. Although it has a similar basis weight to standard Xerox paper, knotweed paper is significantly thicker and less dense, indicating a less compact and more porous structure. This affects moisture and ink absorption, resulting in lower dimensional stability, reduced mechanical resistance, and higher surface roughness. The measured moisture content and high specific volume confirm that knotweed paper has a more open structure compared to the reference papers. Although such a structure provides higher bending stiffness, it reduces the precision of pigment transfer, which was particularly evident in inkjet printing. These results complement existing literature [44,45,46], which indicates that uniform ink application is more difficult to achieve on porous and rough substrates, especially when printing with water-based inks. Our results show that the ΔE values, representing colour deviation, for knotweed paper almost never reach the acceptable thresholds for professional printing (ΔE < 5), confirming that the paper is unsuitable for applications where visual accuracy is critical (e.g., logos and corporate graphic identity). Comparison with the findings of Karlovits et al. (2020) [45] confirms that surface roughness and the yellowish tone of the substrate significantly affect colour saturation, optical gloss, and perceived print quality.

The ageing behaviour of the prints can be directly correlated with the intrinsic properties of the tested papers. Papers with lower whiteness and higher porosity, such as Japanese knotweed paper, exhibited more pronounced colour changes after xenon light exposure, as the yellowish base tone and open fibrous structure amplify perceptible shifts in colour coordinates. Increased surface roughness and moisture content further influence ageing performance by promoting non-uniform toner or ink fixation and accelerating photo-oxidative processes at the paper–ink interface. These results confirm that colour stability during ageing is determined not only by the printing technique but also by the combined interaction between paper optical properties, surface structure, and ink or toner adhesion.

The results of our study, involving printing the logo of DTM Tehnologije d.o.o., support this observation: the red colour of the logo, essential for maintaining brand identity, was reproduced significantly less accurately on knotweed paper, even when ICC profiles were applied. Despite these limitations, the results also confirm certain advantages of knotweed paper, particularly from a sustainability and mechanical perspective. Japanese knotweed paper has greater Clark stiffness, which can be advantageous in packaging applications or other cases where mechanical stability is required. Increased elasticity after UV exposure, as reported by Kapun et al. [12], also raises the question of improved long-term durability, which merits further investigation. From an economic perspective, paper derived from invasive plants presents an interesting alternative, particularly for smaller, agile companies capable of rapidly implementing sustainable practices and adapting corporate graphic identity solutions. In addition to environmental benefits (reducing the biological pressure of invasive species, decreasing the use of wood pulp), such a solution also has potential for niche, high-value products such as unique printed materials, packaging, or promotional items for environmentally oriented brands. Despite attempts at manual ICC profile optimisation and the use of existing standardised solutions, the study indicates that colour management for papers with very low whiteness and high porosity requires more than just profile adjustments—it would necessitate the integration of white underlayers, advanced coating techniques, or the development of entirely new strategies for colour calibration on atypical substrates. This is further supported by the failure of the developed profile (ΔE > 43) and the clearly perceptible colour shifts across the spectrum.

Future research should therefore focus on the following directions:

- Development of coating strategies that improve optical properties without reducing the sustainable value of the paper.
- Testing the use of white primers in inkjet printing, which could significantly improve the reproduction of brighter colours.
- Researching other printing techniques (e.g., UV printing or dry offset) and analysing their compatibility with such substrates.
- Inclusion of user perception studies (evaluation of perceived print quality).

From a broader societal perspective, this study confirms that alternative materials, such as paper from invasive plants, have the potential to be integrated into sustainable value chains—but only if their technical limitations are clearly recognised and adequately considered in practice.

## 5. Conclusions

The main objective of this study was to assess the suitability of paper made from cellulose fibres of the invasive plant Japanese knotweed for colour reproduction in a commercial context. The primary research question concerned the ability to accurately reproduce a company logo—particularly a specific red colour—on paper with low whiteness and a modified surface texture. Over five trial phases, different paper types were compared, printing techniques were tested, a custom ICC colour profile was developed, and colour differences between the reference and actual prints were analysed. The results show that paper made from Japanese knotweed differs significantly from standard papers in its properties. The greater thickness, lower density, and higher moisture content affect its mechanical behaviour, while the porous and rough surface leads to uneven ink application and higher ΔE values, especially in inkjet printing. The best results for colour reproduction were obtained with electrophotography on commercial papers, while the differences observed with inkjet printing on knotweed paper were too great for professional use. From an economic perspective, Japanese knotweed is an interesting raw material for companies seeking acceptable solutions and willing to customise their graphic materials. Despite the technical challenges, paper from invasive plants offers significant potential, as it reduces the use of wood resources while helping to combat the spread of invasive species. For future research, a stronger focus on surface treatment of the paper (e.g., coatings or white paints), the development of specialised colour profiles, and a better understanding of the interactions between printing techniques and non-traditional papers is recommended. It would also be valuable to investigate the use of knotweed paper in applications where mechanical or environmental durability is more important than aesthetic perfection (e.g., packaging, interior materials, promotional printed materials for sustainable brands).

## Figures and Tables

**Table 1:** Thickness, grammage, and density of the tested paper grades.

Paper Grade	Thickness [µm]	Grammage [g/m^2^]	Density [kg/m^3^]
Office	98 ± 1.82	76.9 ± 0.9	782 ± 8.26
Xerox	101 ± 1.22	96.4 ± 0.5	954 ± 12.14
Knotweed	154 ± 3.00	97.1 ± 2.7	630 ± 11.00

**Table 2:** Moisture content and specific volume of the tested paper grades.

Paper Grade	Moisture Content [%]	Specific Volume [cm^3^/g]
Office	2.88 ± 0.13	1.28 ± 0.01
Xerox	3.37 ± 0.11	1.05 ± 0.01
Knotweed	5.7 ± 0.10	1.59 ± 0.03

**Table 3:** Surface roughness and porosity of the tested paper grades (sides A and B).

	Roughness [mL/min]		Porosity [mL/min]	
Paper Grade	A	B	A	B
Office	218 ± 12	279 ± 9	823 ± 58	807 ± 51
Xerox	34 ± 1	33 ± 8	361 ± 17	336 ± 20
Knotweed	1128 ± 91	868 ± 45	401 ± 9	372 ± 23

**Table 4:** Speed of sound (C), modulus of elasticity (E), and Clark stiffness (CS) of the tested paper grades in the machine direction (MD) and cross direction (CD).

	C [km/s]		E [MPa]		CS [Nmm]	
Paper Grade	MD	CD	MD	CD	MD	CD
Office	2.98 ± 0.10	2.33 ± 0.08	6969 ± 469	4234 ± 386	0.55 ± 0.04	0.33 ± 0.03
Xerox	3.54 ± 0.11	2.44 ± 0.13	11,962 ± 1047	5670 ± 384	1.03 ± 0.09	0.49 ± 0.03
Knotweed	3.30 ± 0.16	2.32 ± 0.09	6884 ± 552	3382 ± 250	2.10 ± 0.17	1.03 ± 0.08

**Table 5:** Specular gloss of the tested paper grades for sides A and B in the machine direction (MD) and cross direction (CD).

	A		B	
Paper Grade	MD [%]	CD [%]	MD [%]	CD [%]
Office	3.40 ± 0.20	3.70 ± 0.34	2.90 ± 0.26	3.10 ± 0.24
Xerox	9.30 ± 0.55	10.40 ± 0.52	7.00 ± 0.24	7.90 ± 0.66
Knotweed	4.30 ± 0.26	4.90 ± 0.33	3.70 ± 0.26	5.00 ± 0.31

**Table 6:** Whiteness of the tested paper grades, measured by the R457 D65 method and expressed as CIE W values.

Paper Grade	R457 D65	CIE W
Office	101.69	412.80
Xerox	108.50	156.19
Knotweed	35.96	−52.96

**Table 7:** Colour differences (ΔE*00) of the tested paper grades after artificial ageing for electrophotographic and inkjet printing.

Printing Technique	Paper Grade	ΔE*00
Electrophotography	Office	0.54
	Xerox	0.54
	Knotweed	0.79
Inkjet	Office	1.03
	Xerox	1.37
	Knotweed	0.87

**Table 8:** Colour differences (ΔE*00) of the tested paper grades after rubbing tests at two load levels (12 N and 24 N) in inkjet printing.

Paper Grade	12 N	24 N
Office	3.54	8.10
Xerox	7.98	13.54
Knotweed	9.98	9.78

**Table 9:** CIELAB values and colour difference (ΔE) for the red produced with the custom ICC profile compared to the original red (Xerox–electrophotography).

Reading	L*	a*	b*
Red profile	59.59	10.83	11.35
Red original (Xerox–electrophotography)	29.35	39.63	23.07
ΔE	43.37		

**Table 10:** Colour differences (ΔE) for the tested ICC profiles applied to paper containing Japanese knotweed.

Colour Profile	ΔE
Default ICC profile (Xerox)	27.78 ± 0.17
ISO Uncoated Yellowish	28.15 ± 0.18
Fogra 39 (coated papers)	28.72 ± 0.30
Japan Colour 2002 Newspaper	43.91 ± 0.15

## Data Availability

The original contributions presented in this study are included in the article. Further inquiries can be directed to the corresponding author.

## Abbreviations

The following abbreviations are used in this manuscript:

ΔE*94	Colour difference according to CIE94 formula
ICC	International Colour Consortium
MD	Machine Direction
CD	Cross Direction
CS	Clark Stiffness
ISO	International Organisation for Standardisation
UV	Ultraviolet
CIE	Commission Internationale de l’Éclairage (International Commission on Illumination)
D65	Standard daylight illuminate used in colorimetry
R457	ISO brightness index based on reflectance at 457 nm
